# HMPV-TherResDB: Comprehensive human metapneumovirus (HMPV) database for sequence-structure annotations, vaccine resources, and therapeutics research

**DOI:** 10.1016/j.virusres.2025.199665

**Published:** 2025-11-16

**Authors:** Abbas Khan, Anwar Mohammad, Fahad M. Alshabrmi, Eid A. Alatawi, Muhammad Junaid, Abdelali Agouni

**Affiliations:** a Department of Pharmaceutical Sciences, College of Pharmacy, QU Health, Qatar University, P.O. Box 2713, Doha, Qatar; b Division of Bioinformatics, Department of Biomedical Sciences, Faculty of Medical and Life Sciences, Sunway University, Sunway City 47500, Malaysia; c Precision Health Analysis Unit, Translational Research, Dasman Diabetes Institute, Dasman, Kuwait; d Department of Medical Laboratories, College of Applied Medical Sciences, Qassim University, 51452 Buraydah, Saudi Arabia; e Department of Medical Laboratory Technology, Faculty of Applied Medical Sciences, University of Tabuk, Tabuk 71491, Saudi Arabia; f Institute for Advanced Study, Shenzhen University, Shenzhen 518060, China; g College of Physics and Optoelectronics Engineering, Shenzhen University, Shenzhen 518060, China

**Keywords:** HMPV, Sequences, Structures, Vaccines, Drugs, RNA-therapeutics

## Abstract

Human metapneumovirus (HMPV), a current escalating health issue, causes respiratory complications in young children, the elderly, and immunocompromised individuals. To date, no specific treatment is available; thus, to support vaccine and therapeutic development, we present HMPV-TherRes: a specialized database for HMPV research. This platform integrates a wealth of genomic, proteomic, structural, and immunological data, as well as target-specific drugs, RNA-based therapeutics, and CRISPR-based designs, offering an invaluable resource for advancing both basic and clinical research. The database integrates data generated through state-of-the-art and AI-powered algorithms. The database hosts 618 annotated genomes from various parts of the world, along with protein information, including their physicochemical properties, and experimentally derived or AlphaFold-predicted 3D structures. Moreover, immune resources are a central feature, encompassing detailed information on the predicted and experimentally reported cytotoxic T lymphocyte (CTL) epitopes, helper T lymphocyte (HTL) epitopes (IFN ±), and B-cell epitopes. Additionally, it includes curated datasets, multi-epitope vaccines, and mRNA-based vaccine candidates, underscoring its utility in vaccine design and development. The database also provides data on different drugs targeting hMPV, along with extensive RNA-therapeutic resources, such as siRNAs and miRNAs, which are instrumental in gene-silencing applications. Further expanding its scope, HMPV-TherRes includes CRISPR-based sgRNA designs for both Cas9 and Cas13, enabling targeted genome editing and regulation of the transcriptome. HMPV-TherRes is a versatile repository bridging experimental and computational studies, consolidating diverse resources to support vaccine design, RNA therapeutics, and drug development. It advances understanding of hMPV biology, accelerating efforts to combat the pathogen. This centralized, user-friendly platform represents a significant advancement in virology, enabling researchers to develop novel interventions against HMPV. The database can be accessed through: https://ddd.agounikhanlabs.com/hmpvetherpresdb/index.php.

## 1 Introduction

The human metapneumovirus (HMPV) is the causative agent that was first discovered in 2001 (Van Den Hoogen et al., 2004). It primarily targets children, the elderly, and immunosuppressed individuals, causing bronchiolitis and pneumonia that range from mild to severe, affecting the lower respiratory system. HMPV pathogenesis involves viral replication in the epithelial cells of the upper and lower respiratory tracts, which activates the immune response, accompanied by excessive muscle contraction and secretions that block airflow. The most prevalent symptoms include fever, cough, wheeze, and other forms of breathlessness. Epidemiological data have established HMPV as one of the significant contributing pathogens after the respiratory syncytial virus and rhinovirus (Shafagati and Williams, 2018). The viral burden, coupled with increasing medical help-seeking behaviors, has only contributed to its high healthcare costs. In late December 2024, a growing number of hospitalizations in China and other countries were reported. The USA, UK, and Australia are among the rising regions with a critical prevalence (Feng et al., 2024). Currently, there is no definitive antiviral treatment for HMPV; however, oxygen supplementation is recommended. The increasing incidence demands a thorough investigation of the genomic and proteomic features of this virus to develop a treatment strategy to overcome the burden of any potential pandemic in the future. The use of computational approaches is very significant in this regard.

Biological databases play a critical role in storing, organizing, and providing access to vast amounts of biological data, including genomic, proteomic, structural, and pharmacological information (Baxevanis and Bateman, 2015; Zou et al., 2015). In particular, developing therapeutic databases primarily focused on data related to vaccine design, drug discovery, and other therapeutic interventions is very advantageous (Schneeweiss and Avorn, 2005). The role of such databases is indispensable in managing infectious diseases by providing easy access to curated information related to viral, bacterial, or any other microbial genomes, druggable proteins, and immune epitopes essential for vaccine design (Phadke et al., 2021). For instance, the Influenza Research Database (IRD), dedicated to influenza viruses, and the COVID-19 Data Portal expedite research on these viruses by integrating diverse data sequences, structural data, and drugs. Similarly, the Comprehensive Antibiotic Resistance Database supports research on combating antibiotic resistance (McArthur et al., 2013). Moreover, other databases, such as HantavirusesDB and MMV-db, integrate data on sequences and vaccines from Hantaviruses and mammarenaviruses (Khan et al., 2021; Khan et al., 2021). These repositories facilitate the analysis of complex datasets and potentially lead to the discovery of novel therapeutic strategies, which in turn can aid in the development of targeted vaccines, RNA-based therapeutics, and CRISPR-based interventions, thereby advancing global health initiatives against these pathogens.

To address the critical need for a comprehensive resource dedicated to HMPV, we developed HMPV-TherResDB, a specialized database designed to support vaccine and therapeutic research. This platform is essential due to the growing burden of hMPV as a global health concern, as well as the lack of specific treatments and vaccines. By consolidating diverse datasets, including genomic, proteomic, structural, immunological, and therapeutic information, HMPV-TherResDB provides a centralized resource for researchers to explore the biology of hMPV and develop targeted interventions. We aim to bridge the gap between experimental and computational research, enabling the discovery of novel therapeutics, RNA-based therapies, and vaccine candidates. Ultimately, HMPV-TherResDB seeks to enhance the understanding of hMPV pathogenesis and expedite the development of effective strategies to combat this pathogen, thereby making significant contributions to the fields of virology and public health.

## 2 Materials and methods

### 2.1 Data retrieval and curation

**Fig. 1:**
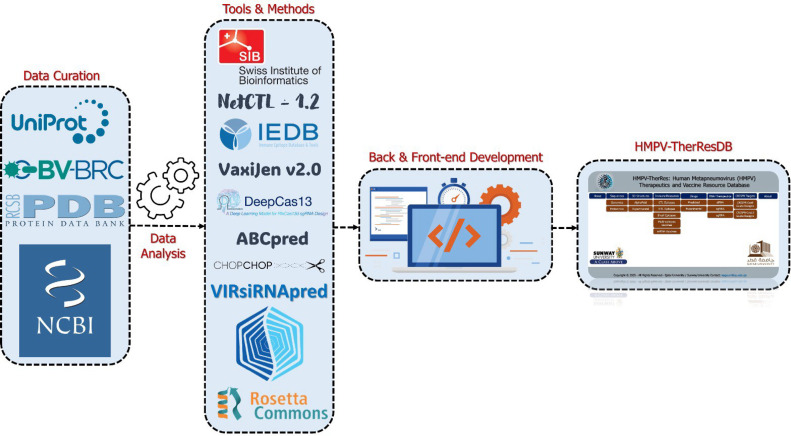
Systematic workflow of the data acquisition and analysis using different computational and AI-powered algorithms. The workflow consists of four steps: data retrieval and preparation, Data analysis using various algorithms, back-end and front-end development, and finally, deployment of the data for users.

For the construction of the HMPV therapeutic database, genomic and protein information were curated from various reliable sources. For instance, UniProt (https://www.uniprot.org) was used to retrieve the protein sequences in FASTA format (accession number: UP000001398), which contain rich annotations. Meanwhile, the NCBI (https://www.ncbi.nlm.nih.gov) was used for genomic data (accession number: AY297749.1), and the BV-BRC (https://www.bv-brc.org) provided additional resources. Whole-genome sequences were also collected from diverse countries to ensure both geographic and complete diversity. For the experimentally available 3D structures, we used RCSB (https://www.rcsb.org/) (Burley et al., 2019). All the collected data were manually curated to remove redundancy, standardized, and ensured to produce high-quality, error-free sequences, which were then used for downstream processing. Fig. 1

### 2.2 Annotation of the HMPV whole proteome

Protein sequences retrieved from UniProt were analyzed to predict the essential physicochemical and immunogenic properties. For this purpose, ProtParam (https://web.expasy.org/protparam/), a web server, was utilized to estimate basic protein features, such as molecular weight, isoelectric point, aliphatic index, and instability index (Artimo et al., 2012). Antigenicity predictions. On the other hand, we evaluated the antigenic properties of each protein using VaxiJen (http://www.ddg-pharmfac.net/vaxijen/VaxiJen/VaxiJen.html). This tool utilizes alignment-dependent methods to determine the antigenicity of each protein, thereby inducing an immune response (Doytchinova and Flower, 2007). Furthermore, allergenicity was assessed using the AllerTOP (https://www.ddg-pharmfac.net/AllerTOP/) and AllergenFP (http://ddg-pharmfac.net/AllergenFP/) methods (Dimitrov et al., 2013; Dimitrov et al., 2014). This information is essential for understanding the patterns of the HMPV proteome that could help select an appropriate vaccine candidate.

### 2.3 AlphaFold 3.0 guided structural modeling

Three-dimensional structures of the chosen proteins were obtained using the recently released AlphaFold 3 (https://www.alphafold.ebi.ac.uk/), the most advanced AI-based approach for protein structure prediction (Abramson et al., 2024). AlphaFold 3 uses advanced deep learning methods to model 3-dimensional protein structures with remarkable precision. This involves a neural network trained on enormous collections of sequence and structural data to recognize the patterns and connections established between amino acids. The process goes one step further, with the model iteratively improving its predictions based on spatial and chemical information to produce 3D structures of high confidence that often rival experimental methods for precision. The pLDDT score of each protein serves as a quality metric to determine the prediction accuracy of each residue.

### 2.4 Identification and mapping of immunogenic epitopes in the HMPV’s proteome

We predicted Cytotoxic T Lymphocytes (CTLs), Helper T Lymphocytes (HTLs), and B cells for each protein to be included in the database to assist in the vaccine design process. The CTL epitopes were predicted based on the cleavage of the protein into fragments by the proteasome, the transport efficacy of these fragments into the endoplasmic reticulum by TAP, and their binding affinity with MHC class I molecules using the NetCTL server (https://services.healthtech.dtu.dk/service.php?NetCTL-1.2) (Larsen et al., 2007). The IEDB server (http://tools.iedb.org/mhcii/) was used to predict HTL epitopes by evaluating binding to the MHC class II molecule (Vita et al., 2019). The interferon (IFN)-gamma-inducing capacity of these HTL epitopes was further determined from the IFN-epitope server (http://crdd.osdd.net/raghava/ifnepitope/), and only IFN-positive epitopes were considered (Dhall et al., 2024). B-cell epitopes were predicted through ABCpred (http://crdd.osdd.net/raghava/abcpred/) with a stringent threshold of 0.90 (Malik et al., 2022). Other experimentally validated CTL epitopes were also collected from BV-BRC (https://www.bv-brc.org) and added to enrich the dataset.

### 2.5 Optimized multi-epitope vaccine constructs deployment

Multi-epitope vaccine constructs (MEVCs) are pivotal for addressing infectious diseases, as they can be customized to elicit targeted immune responses against diverse pathogens. Using the predicted CTL, HTL, and B-cell epitopes, we designed MEVCs for each protein separately by combining suitable linkers and adjuvants to enhance immunogenicity. We also designed a final composite MEVC consisting of all proteins' epitopes. We used an optimization strategy by supplying different adjuvants and linkers. The incorporation of different adjuvants, linkers, and epitope combinations is a crucial strategy for optimizing multi-epitope vaccines by enhancing their immunogenicity and efficacy. Adjuvants boost the immune response, linkers ensure proper epitope presentation, and tailored epitope combinations target specific immune pathways for robust and durable protection. The constructs were evaluated for antigenicity and allergenicity using VaxiJen (http://www.ddg-pharmfac.net/vaxijen/VaxiJen/VaxiJen.html) and AllerTOP (https://www.ddg-pharmfac.net/AllerTOP/), respectively. Moreover, physicochemical properties, including molecular weight, isoelectric point, and stability, were evaluated using ProtParam (https://web.expasy.org/protparam/). We used the Robetta server (https://robetta.bakerlab.org/) for the structural modeling of MEVC (Kim et al., 2004). The Robetta server is one of the best methods for Ab-initio modeling to 3D model the vaccine. Robetta offers a hybrid approach to de novo structure prediction by incorporating template-based modeling and producing models derived from amino acid sequences and, when available, homologous templates. It builds and evolves models as it assembles fragments according to physical and statistical energy functions. Such techniques are particularly useful with new or complex sequences, as they provide structures that can be critical in understanding how vaccines fold, function, and interact with immune targets. The designed MEVCs were optimized for efficient expression in the host system through codon optimization by using VectorBuilder (https://en.vectorbuilder.com/). The optimized sequences, along with their GC content, codon adaptation index (CAI), and secondary structure stability, are available in the database to verify their suitability for experimental verification. Including MEVCs in therapeutic databases accelerates vaccine research by providing comprehensive data on design, efficacy, and molecular interactions, facilitating the rapid development of effective vaccines for emerging and re-emerging infectious threats.

### 2.6 Exploring the chemical space for HMPV-targeted inhibitors

HMPV target-specific lead molecules were also identified and deployed online for therapeutic purposes. We retrieved the entire South African Natural Compounds database (SNCDB) in “.sdf” format. We utilized our in-house Python scripts, which incorporate RDKit and OpenBabel with Biopython modules, to filter the database based on R5 rules and prepare the compounds by minimizing and adding hydrogenatoms. Among the total of 1200 compounds, only 645 met the criteria and were included in the screening step. We employed a three-tiered virtual screening protocol using the Schrödinger Maestro software, which incorporates high-throughput screening (HTS), standard precision (SP) docking, and extra precision (XP) docking procedures. For each protein, preparation and minimization steps were performed, and the active sites were defined either based on the literature or using the Sitemap tool in the Schrodinger package. The top 10 hits from each target were added to the database, which contains information regarding the database accession number, 2D structures, SMILES, scientific names, and docking scores. The protocol ensured that high-affinity binders and specific binders appropriate for therapeutic use were identified.

### 2.7 RNA designs and CRISPR-Cas9/13 targets prediction

Small interfering RNA (siRNA), single-guide RNA (sgRNA), and microRNA (miRNA) were designed to target specific portions of the HMPV genome with the intention of gene silencing and therapeutic applications. Mapping the siRNAs in the genome of the HMPV virus is a crucial step towards RNA silencing, which could help eliminate the virus from the host. For this purpose, we utilized the VIRsiRNApred (http://crdd.osdd.net/servers/virsirnapred/) server, which employs an artificial intelligence-guided approach to map potential siRNAs throughout the entire genome, providing precise locations and inhibition potential. The inhibitory potential of 50 % was set to include the siRNAs in the database, since the others may not effectively silence the genes. For miRNA mapping, a standalone package of VMir was downloaded and installed on the local server (Grundhoff, 2011). The package contains both VMir Analyzer and VMir Visualizer, which were subsequently used to identify the precursor miRNAs. The entire genome was uploaded to the VMir analyzer, which successfully mapped the potential miRNAs in the HMPV genome. The determined miRNAs were then visualized through the VMir visualizer. Since these tools only provide information regarding location and other scorings, we used an in-house Python script to precisely map the sequences of these miRNAs across the entire genome, based on the information obtained from VMir software. The CRISPR systems are useful as anti-viral therapeutics as CRISPR-Cas9/Cas13 have a much higher therapeutic potential due to their ability to target a range of viral pathogens based on their structures. Cas9 can be programmed to bind to specific regions in viral DNA and disrupt it, thereby preventing replication in DNA viruses. In contrast, Cas13 is specifically applicable against viral RNA, making it suitable for use against RNA viruses. Their high specificity minimizes off-target effects, and they can be engineered to target conserved viral regions, enhancing broad-spectrum antiviral potential. These properties position Cas9 and Cas13 as promising platforms for developing next-generation antiviral treatments, particularly for emerging and drug-resistant viral infections. While Cas9 primarily targets DNA, it can be adapted for RNA viruses using engineered variants. The CRISPR system Cas9 and Cas13 target sites were identified using the web tools CHOPCHOP (https://chopchop.cbu.uib.no (Labun et al., 2019) and CRISPRscan (https://www.crisprscan.org (Moreno-Mateos et al., 2015). To determine the efficiency of CRISPR-cas13 guided sgRNA, we used the DeepCas13 web server (http://deepcas13.weililab.org/ (Cheng et al., 2023). The specificity and feasibility of the targets were evaluated for the application of gene editing and antiviral treatment.

### 2.8 Front-End and back-end development

The user interface of the database was developed using HTML (https://html.com), CSS (https://developer.mozilla.org/en-US/docs/Web/CSS), and JavaScript (https://developer.mozilla.org/en-US/docs/Web/JavaScript) to provide an accessible user experience and ensure cross-platform compatibility. The web pages were arranged using HTML, while CSS made them more visually appealing and adaptive in layout. JavaScript added interactivity to the database through search filters and real-time changes. The backend was developed on WordPress (https://wordpress.org) as the CMS, which enables good data management. The server-side logic was developed using PHP (https://www.php.net) to handle database queries efficiently. MySQL (https://www.mysql.com) was used as the database management system to store and retrieve genomic, proteomic, and epitope data. APIs were also created to enable integration with external tools and datasets while providing secure user access with efficient data processing speed. This architecture supported a reliable, accessible, and dynamic HMPV database.

## 3 Results and discussion

### 3.1 Interface of HMPV-TherResDB database

**Fig. 2:**
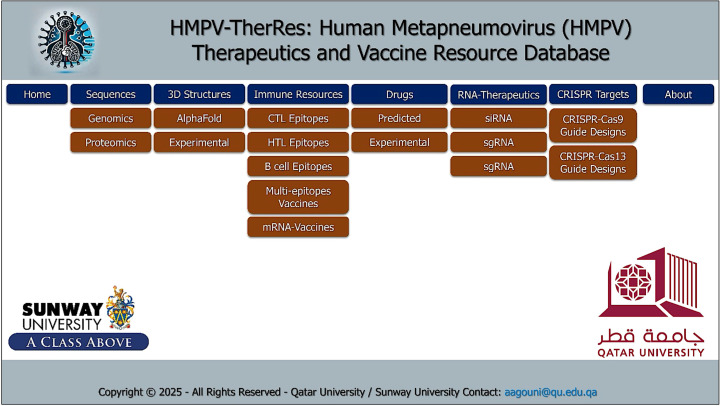
The interface design of HMPV-TherResDB, which users can use to retrieve specific information.

HMPV virus is currently an escalating global health issue, with the number of incidences steadily increasing since last month in different parts of the world. Since no specific treatment is available for HMPV virus infection, efforts are needed to address this emerging global health issue. Therefore, we developed “HMPV-TherResDB,” which will serve as a cornerstone in shaping the treatment and clinical diagnosis of HMPV virus-caused infections. This repository is the first to provide comprehensive information, from raw sequences to CRISPR-based treatments. The interface is designed to provide a systematic way to access specific and related information in each tab. A total of nine tables which includes the front “Home”, “Sequences” which further contain genomics and proteomics sub-tabs, “3D structures”, “Immune Resources” which has further specific sub-tabs, “Drugs”, “RNA-Therapeutics”, “CRISPR Targets” for cas9 and cas13 systems, “Downloads” and finally “About” tabs are available to access different information related to HMPV virus. The interface design is shown in Fig. 2.

### 3.2 Data statistics

The HMPV-TherResDB is the only repository to support hastening research by providing access to diverse, essential data that can be used to derive meaningful clinical outcomes. The HMPV-TherResDB database serves as a comprehensive resource for advancing research on human metapneumovirus (hMPV) by providing a wide array of genomic, proteomic, structural, and therapeutic data. It includes 618 annotated genomes and nine core proteins, with corresponding 3D structures also cataloged. The immune resources encompass diverse epitope data, including 324 predicted and experimental CTL epitopes, 340 HTL epitopes classified as IFN-positive (117) and IFN-negative (223), and 81 B-cell epitopes, alongside curated information on 34 multi-epitope vaccines (MEVCs) and 10 mRNA vaccines. Furthermore, the database features 70 drugs targeting hMPV and extensive RNA-therapeutics data, with 134 siRNAs and 274 miRNAs. It also provides a robust collection of CRISPR-based sgRNAs, including 866 Cas9 and 10,709 Cas13 sgRNAs, for genome editing applications. This repository offers a critical resource for leveraging computational and experimental approaches to support the development of vaccines, therapeutics, and novel interventions against hMPV. The data statistics are given in Table 1.

**Table 1:** Data statistics for the deployed resources related to the HMPV virus.

HMPV-TherResDB Database Resources and Statistics	
Genomes	618
Proteins	9
3D Structures	9
Immune Resources	
CTL Epitopes Predicted Experimental HTL Epitopes IFN-positive IFN-negative B cell Epitopes Multi-Epitopes Vaccines (MEVCs) mRNA-Vaccines	324 101 223 117 36 81 34 10 10
Drugs for HMPV	70
RNA-Therapeutics	
siRNAs miRNAs	134 274
CRISPR-based sgRNAs for cas9/13	
Cas-9 based sgRNAs Cas-13 based sgRNAs	866 10,709

### 3.3 Utility of HMPV-TherResDB

**Fig. 3:**
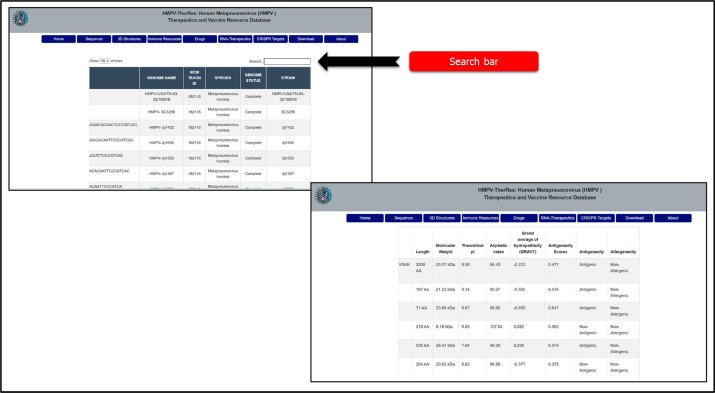
Demonstrates the utility of genomics and proteomics in HMPV. The figure provides information on the contents of each sub-tab and its application.

The utility of this database lies in its unparalleled capacity to serve as a comprehensive and centralized hub for advancing both basic and applied research on HMPV. By providing detailed genomic and proteomic data, it empowers researchers to decode the molecular intricacies of HMPV, paving the way for precision-targeted interventions. The Sequences tab offers comprehensive information on genomes, locations, strain details, NCBI GeneBank accession numbers, and other essential details. Researchers can download complete sequences or region-specific ones, such as country or continent, which can be used to reconstruct the evolutionary path and understand the transmission dynamics. Moreover, information regarding protein sequences can be retrieved by clicking the Proteins sub-tab in the sequences, where all information, from sequence to physicochemical properties and immunogenic properties, is also available. Furthermore, a search bar is provided on each sub-tab, allowing users to search for specific information based on name, target, sequence, location, year, and other parameters listed on the particular page. This information is visualized in Fig. 3.

**Fig. 4:**
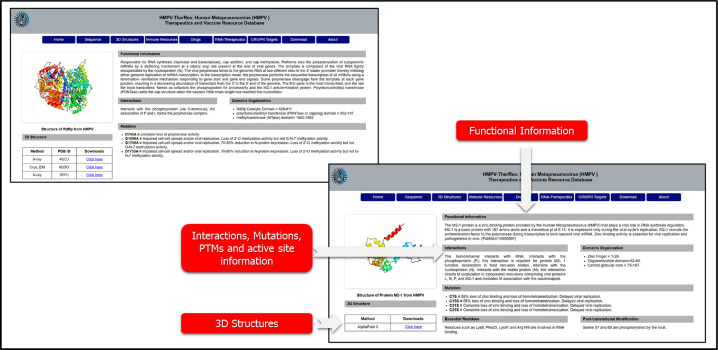
Shows the utility of the 3D structures tab of HMPV. The figure provides the functional information, mutational data, PTM, PPIs, and 3D structures.

Its inclusion of 3D structural models, powered by AlphaFold and experimental studies, enables the design of particular inhibitors and drugs. The 3D structures tab can be used to assess specific information regarding each protein in the HMPV. Upon accessing each sub-tab, the database provides information on the function of a protein, its 3D structure, interactions with other proteins, post-translational modification sites, and reported mutations. Moreover, this tab also provides access to the available 3D structures, whether x-ray, cryo-EM, or resolved through AlphaFold 3.0. Users can download a specific structure with a single click and can be used in subsequent research. An example usage of the 3D structure tab is shown in Fig. 4.

**Fig. 5:**
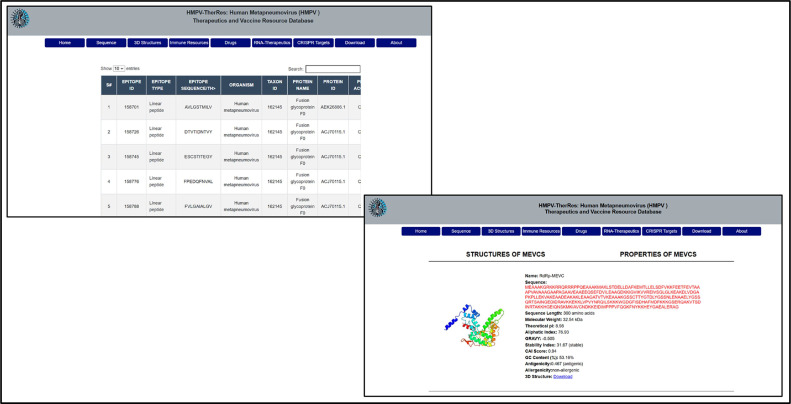
Shows the utility of the Immune Resources tab of HMPV. The figure provides epitope sequences, MEVCs, 3D structures, and physicochemical properties, including the CTL, HTL, and B cell epitopes, in the data repository.

The immune resource section, featuring epitopes and vaccine constructs, is a cornerstone for developing innovative vaccine candidates, particularly multi-epitope and mRNA-based vaccines, which hold immense potential for inducing robust immunity in vulnerable populations. The Immune resources tab gives access to users to retrieve information regarding the potential available epitopes in the proteome of HMPV, whether CTL, HTL, or B cell epitopes, with their specific parameters. More importantly, these epitopes are already filtered with stringent criteria to add highly immunogenic and potential epitopes that researchers can use with their own defined adjuvants and linkers to design multi-epitopes or can be used alone to trigger the immune response in experimental models. To further facilitate access, we have developed our own MEVC and deployed it online in the multi-epitopes vaccine (MEVC) sub-tab, which provides information on the sequence of each MEVC, its physicochemical properties, antigenicity, allergenicity, CAI index, GC content, and the 3D structure of MEVC. The user can directly retrieve the sequence, which is also supplied with highly effective adjuvants, linkers, and His-tags, and can modify or use it as needed. For each protein, a MEVC was designed and added, and a consensus MEVC is also provided. The sequences of mRNA vaccines can also be accessed through this tab. The specific usage is shown in Fig. 5.

**Fig. 6:**
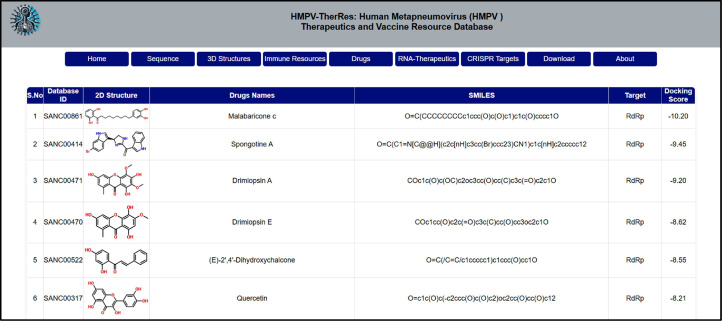
Shows the utility of the Drugs tab of HMPV. This tab provides access to information such as database ID, scientific names, SMILES, 2D structures, target information, and, finally, the docking scores against each drug.

The drug information is also supplied in the “Drugs” tab, which researchers can use to retrieve specific information regarding potential drugs against each target. This tab provides users with access to the drugs that are reported to be the most effective in the entire database. The database ID, scientific names, SMILES, 2D structures, target information, and finally the docking scores against each drug are provided. This functionality can be used to identify similar drugs in other databases or to directly test these drugs experimentally. This will add value to expedite further HPMV treatment research, while also focusing on the development of the vaccine. The utility of this tab is shown in Fig. 6.

**Fig. 7:**
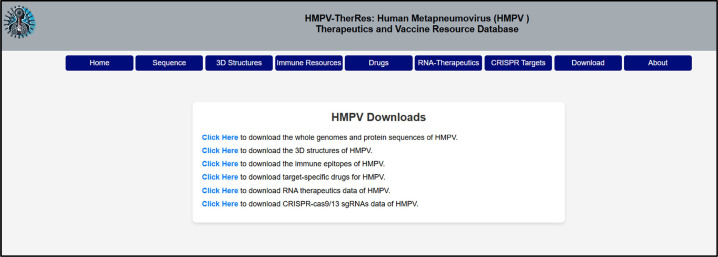
Shows the utility of the Downloads tab of HMPV. This tab gives access to download all the data in raw form.

Moreover, the RNA-therapeutics and CRISPR-targeting tools are revolutionary, offering researchers cutting-edge technologies for gene silencing and genome editing to curb viral replication and modify host susceptibility. The “Downloads” tab (Fig. 7) provides users with access to download all the raw data generated through state-of-the-art computational methods and use it for various other analyses. Clinicians and pharmaceutical developers can leverage the drug repository to identify and validate novel antiviral compounds, speeding up the path from bench to bedside. This database serves as a bridge between fundamental science and clinical application, providing a one-stop resource for accelerating the development of therapeutic and vaccine options against HMPV, ultimately contributing to global efforts in combating respiratory infections.

### 3.4 Applications of HMPV-TherResDB

This state-of-the-art HMPV-TherResDB database for vaccines and other therapeutics research represents a breakthrough in advancing the treatment choices for HMPV. The genomics and proteomic data provided in the repository offer revolutionary insight into the molecular mechanisms behind pathogenesis and empower researchers with countless opportunities for targeted drug discovery. For instance, genomic information has been successfully used to reconstruct the phylogenetic relationship and understand the transmission routes of different pathogens (Ciccozzi et al., 2019; Lam et al., 2010; Cook and Holmes, 2006; Dennis et al., 2014). The integration of structural data from AlphaFold 3.0 and experimental structural data appears to enable researchers to study the interactions among viral proteins, determine the impact of variations in protein sequence on protein structure and function, and create a gold mine for potentially pinpointing drug-binding sites. Data from such methods are very transformative in advancing the treatment of various diseases (Weng et al., 2022; Johansson-Åkhe and Wallner, 2022; Baselious et al., 2023; Khan et al., 2023). The immunological resources, such as CTL, HTL, and B-cell epitopes, can be utilized to map potential multi-epitope and effective mRNA vaccine designs that can instigate robust and long-acting immune responses to eliminate viral infections caused by HMPV. For instance, such predictions are highly successful in stimulating the immune response in experimental models (Cheng et al., 2023; Kaushik et al., 2022; Cheng et al., 2022; Khan et al., 2022; Junaid et al., 2019). On the drug-discovery platform, a composite repository of both predictive and experimental drugs specific to each target in the HMPV proteome will accelerate the development of life-saving antivirals. Virtual screening methods are considered the cornerstone of identifying and developing new drugs for various diseases. This approach is accurate, fast, and inexpensive for the discovery of novel drugs. For instance, it has been applied to multiple computational and experimental models for the discovery of a diverse set of target-specific ligands (Khan et al., 2024; Tai et al., 2023; Khan et al., 2022; Berry et al., 2015; DiFrancesco et al., 2023). The RNA-therapeutics aspect, with siRNA and sgRNA designs, promises to create guidelines for how siRNA-mediated acute inhibition of viral replication might work. These designs have proven experimental roles, such as siRNAs, which have been used against the Hepatitis C Virus to target conserved regions as well as the ORF1ab of MERS-CoV. Meanwhile, mRNAs are reported to work efficiently in West Nile virus (WNV), SARS-CoV-2, and Dengue virus (DENV) (Sohrab et al., 2021; ElHefnawi et al., 2016; Avila-Bonilla and Salas-Benito, 2024; Shi et al., 2014; Hasan et al., 2021).

The CRISPR-Cas9 and Cas13, on the other hand, are at the forefront of genome editing. Guide designs provide an exceedingly effective toolbox that heralds a new era of precision virology, enabling the disablement of viral genes or expression in related hosts with the highest accuracy. These approaches also have a well-validated track record against various pathogens (Hussein et al., 2022; Zahedipour et al., 2024; Guo et al., 2021). Considering the entire scenario and experimental reports related to the deployed data, this database, clinically, has the potential to expedite therapeutic development routes, enabling revolutionary treatments. In research, it sets the stage for a tidal wave of innovations that will redefine our fight against HMPV. It is not merely a database—it is a game-changer, a beacon of hope, and a critical cornerstone in the global mission to eradicate this pervasive respiratory virus.

## 4 Conclusion

In conclusion, this is the first HMPV-specific data repository to gather information on the diverse aspects of the HMPV virus. The current global situation of HMPV demands a composite repository to support therapeutic research powered by computational and AI methods. The utility of this database lies in its unparalleled capacity to serve as a comprehensive and centralized hub for advancing both basic and applied research on HMPV. Considering the whole scenario and experimental reports related to the deployed data, this database, clinically, has the potential to expedite therapeutic development routes, enabling clinically safe and effective treatments. In research, it sets the stage for a tidal wave of innovations that will redefine our fight against HMPV. It represents a comprehensive and integrative bioinformatics resource that consolidates multi-dimensional therapeutic and immunological data, providing a robust platform to accelerate research and rational design strategies aimed at combating Human Metapneumovirus.

## CRediT authorship contribution statement

**Abbas Khan:** Writing – review & editing, Writing – original draft, Visualization, Methodology, Investigation, Formal analysis, Data curation, Conceptualization. **Anwar Mohammad:** Investigation, Funding acquisition, Formal analysis, Data curation, Conceptualization. **Fahad M. Alshabrmi:** Investigation, Funding acquisition, Formal analysis, Data curation, Conceptualization. **Eid A. Alatawi:** Methodology, Investigation, Formal analysis, Data curation, Conceptualization. **Muhammad Junaid:** Investigation, Funding acquisition, Formal analysis, Data curation, Conceptualization. **Abdelali Agouni:** Writing – review & editing, Writing – original draft, Validation, Supervision, Software, Resources, Formal analysis, Data curation, Conceptualization.

## Declaration of competing interest

Declared None.

## Data Availability

All raw data and analysis results are deposited online in the database and are accessible in the Downloads tab.
